# Ecofriendly Flame-Retardant Polystyrene Composites: Exploiting the Synergistic Effects of Phytic Acid, Polyethyleneimine, and Expandable Graphite

**DOI:** 10.3390/ma18184308

**Published:** 2025-09-14

**Authors:** Zhunzhun Li, Qimei Zhang, Jian Cui, Yehai Yan

**Affiliations:** 1 Key Lab of Rubber-Plastics, Ministry of Education/Shandong Provincial Key Lab of Rubber-Plastic, School of Polymer Science and Engineering, Qingdao University of Science and Technology, Qingdao 266042, China; lizz@czu.edu.cn; 2 Anhui Engineering Research Center of Highly Reactive Micro-Nano Powders, School of Materials and Environmental Engineering, Chizhou University, Chizhou 247000, China; zhangqm@czu.edu.cn; 3 Key Laboratory of Micro-Nano Powder and Advanced Energy Materials of Anhui Higher Education Institutes, Chizhou University, Chizhou 247000, China

**Keywords:** polystyrene composite, flame retardant, ecofriendly, synergistic effect, phytic acid, polyethyleneimine, expandable graphite

## Abstract

Ecofriendly flame-retardant polystyrene (PS) composites were developed using the synergistic effects of phytic acid (PA), polyethyleneimine (PEI), and expandable graphite (EG). PA was chemically hybridized with PEI, and the hybrid (PAE) was incorporated into PS together with EG. The flame-retardant performances of the resulting composites were evaluated using the limiting oxygen index (LOI), UL-94 vertical burning test, and cone calorimetry test. The strong interaction between EG and PAE provided an effective barrier against heat and oxygen, thereby improving the flame retardancy. The best-performing composite (PA:PEI:EG = 1:1:1 (*w*/*w*/*w*), total flame-retardant loading = 10 parts per 100 parts of PS) exhibited an LOI of 27.7% and a UL-94 V-0 rating. The peak heat release rate (148.8 kW/m^2^) and total heat release (91.2 MJ/m^2^) of this composite were lower than those of pure PS by 79.2% and 34.0%, respectively. This study provides guidelines for the production of flame-retardant PS and other polymeric materials.

## 1. Introduction

The frequent occurrence of major fires worldwide has drawn considerable attention to environmental protection and fire-resistant material development [1,2]. Polystyrene (PS), which is widely used in civil fields (e.g., electronic materials, packaging, and construction), is highly flammable and poses a substantial fire hazard. Therefore, the flame-retardant properties of PS should be enhanced [3]. Halogenated flame retardants offer high efficiency and are the main industrial flame retardants used in PS. However, these compounds release toxic gases during combustion and pose environmental hazards; this has triggered increasing concerns about their sustainability and prompted extensive domestic and international research on halogen-free flame retardants [4]. Owing to their low toxicity and high performance, phosphorus-based flame retardants have emerged as important alternatives to their halogenated counterparts, and have been extensively applied to various polymers [5]. For instance, Li et al. [6] prepared flame-retardant PS using (3-aminopropyl)triethoxysilane-modified aluminum hypophosphite (AHPi), showing that this compound exhibited flame-retardant effects in both condensed and gas phases. At a modified AHPi content of 25 wt%, the peak heat release rate (PHRR) and total heat release (THR) of the PS composite were lower than those of pure PS by 81.4% and 37.6%, respectively, and a V-0 rating was achieved in the UL-94 vertical burning test (UL-94v test).

As a nonpolar polymer, PS does not readily form a protective char layer during combustion due to the lack of functional groups in its molecular structure that can promote char formation. Instead, it primarily undergoes thermal cracking to generate a large amount of flammable gases. This necessitates the incorporation of char-forming components into the flame-retardant system to enhance its condensed-phase flame retardancy. Wang et al. [7] synthesized a phosphorus–nitrogen flame retardant (PON) using chlorodiphenylphosphate and piperazine. This PON was combined with expandable graphite (EG) to form an intumescent flame retardant for PS. The combination of PON and EG promoted the formation of a protective barrier layer and suppressed the rapid thermal degradation of PS.

Despite these advancements, some commonly used organic phosphorus-based flame retardants pose environmental and health risks [8,9]. Moreover, the phosphorus used in such flame retardants is a nonrenewable resource primarily extracted from phosphate ores. Therefore, sustainable and abundant phosphorus sources for environmentally friendly phosphorus-based flame retardants are urgently required [10]. Among the major phosphorus-containing biomass materials, phytic acid (PA), which is extracted from plant seeds such as grains and soybeans, is a promising candidate. The PA molecule contains six phosphate groups and has a phosphorus content of ~28 wt% [11]. Upon thermal decomposition, PA releases a large amount of phosphoric acid derivatives, which not only promote the dehydration and charring of char-forming agents but also scavenge free radicals, thereby enhancing the overall flame retardancy [12,13,14,15]. As a renewable phosphorus source, PA holds promise for the development of environmentally friendly flame retardants [16,17,18,19]. When used alone as a flame retardant, PA exhibits several drawbacks, including hygroscopicity, poor compatibility with polymers, and insufficient flame retardancy. To overcome these limitations, researchers have explored the synergistic combination of PA with other materials to achieve the desirable flame-retardant performance in PS. For example, Guo et al. [20] synthesized a flame retardant (PAUCG) by reacting PA and urea followed by compounding with modified graphene and multiwalled carbon nanotubes using dopamine as a binder. The EPS-PAUCG composite, fabricated via a coating method, formed a dense intumescent carbon layer during thermal decomposition and achieved a UL-94 V-0 rating.

Currently, PA has achieved satisfactory flame-retardant performances in various polymers, including textiles [21,22,23,24], poly(lactic acid) [25,26,27], polypropylene [28,29,30], and polyurethanes [31,32,33,34]. However, the application of PA as a flame retardant in PS is underexplored. A search on ScienceDirect using the terms “phytic acid and polystyrene” in the title, abstract, or author-specified keywords revealed only six publications on PA flame retardants for PS foam [20,35,36,37,38,39]. Therefore, further in-depth studies are needed to develop and elucidate the flame-retardant mechanisms of PA-based environmentally friendly flame retardants for PS, particularly PS plastic.

In this study, PA was chemically hybridized with polyethyleneimine (PEI), and the resulting hybrid (PAE) and EG were introduced into PS through physical blending. The selection of these materials was driven by their complementary properties and synergistic effects, which collectively enhance the flame-retardant performance of PS composites. PA provides a sustainable phosphorus source, PEI offers strong interfacial interactions and thermal stability, and EG contributes to the formation of a protective barrier. The resulting composites were evaluated in terms of their morphology and limiting oxygen index (LOI) and subjected to thermogravimetric analysis (TGA) and the cone calorimeter test (CCT). Compared with PS, the composites exhibited a higher flame retardancy owing to the formation of a protective carbon layer and reduced heat release rate (HRR). This study provides a scientific basis and technical support for the development of high-performance ecofriendly flame-retardant PS composites.

## 2. Materials and Methods

### 2.1. Materials

PS powder (GPPS-80, 300 mesh) was purchased from Dongguan Hongcheng Plastic Material Co., Ltd. (Dongguan, China). PA (70 wt% aqueous solution) was sourced from Laiyang Wanjiwei Bioengineering Co., Ltd. (Laiyang, China). PEI (Mw = 70,000, 50 wt% aqueous solution) was obtained from Shanghai Macklin Biochemical Co., Ltd. (Shanghai, China). EG (100 mesh) was acquired from Lingshou County Jinyuan Mining Processing Plant (Shijiazhuang, China). All materials were used as received without further purification.

### 2.2. Preparation of Flame-Retardant PS Powders

**Figure 1:**
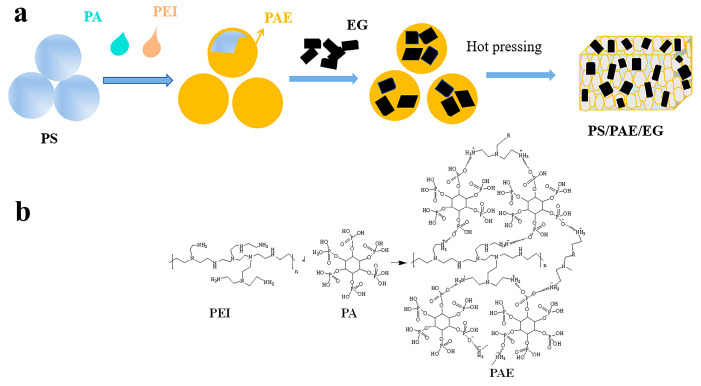
Schematic preparation of the (**a**) PS composites and (**b**) phytic acid (PA)—polyethyleneimine (PEI) flame retardant (PAE).

The employed formulations are provided in Table 1. PS powder was placed in a mortar, supplemented dropwise with the PEI solution, ground for 10 min, supplemented with PA, and further ground for 10 min to obtain PS/PAE powders. To prepare PS/PAE/EG samples, EG was added, and grinding was continued for 10 min. PS/EG samples were obtained by mixing PS and EG in a mortar for 10 min. The samples were dried and stored for further use. The preparation of PS composites and PAE is shown in Figure 1a and 1b, respectively. The ecotoxicity of PAE on the growth of Chlorella vulgaris is provided in the Supplementary Materials.

### 2.3. Characterization

Fourier transform infrared (FTIR) spectra were recorded on a Nicolet iS10 spectrometer (Thermo-Nicolet, Thermo Fisher Scientific, Waltham, MA, USA) using KBr pellets; each sample was scanned 32 times within a wavelength range of 400–4000 cm^−1^.

TGA (HTG-4, Beijing Hengjiu Experimental Equipment Co., Ltd., Beijing, China) was performed at a heating rate of 10 °C/min in air for samples preheated at 150 °C for 5 min.

The sample morphologies were analyzed using scanning electron microscopy (SEM; Phenom ProX Desktop, Phenom World Co., Ltd., Eindhoven, The Netherlands; and TESCAN MIRA LMS, TESCAN, Brno, South Moravian Region, Czech Republic).

The LOI was measured using a TTech-GBT2406 oxygen index meter (TESTech Instrument (Suzhou) Technologies Co., Ltd., Suzhou, China) according to GB/T 2406.2-2009 [40] using specimen dimensions of 100 mm × 10 mm × 4 mm.

The UL-94v test was performed using a TTech-GBT2408 UL94 horizontal vertical combustion tester (TESTech Instrument (Suzhou) Technologies Co., Ltd., Suzhou, China) according to GB/T 2408–2021 [41] using specimen dimensions of 125 mm × 13 mm × 4 mm.

The CCT was conducted using a PX07007 cone calorimeter (Suzhou Phoenix Instrument Co., Ltd., Suzhou, China) at a heat flux of 35 kW/m2 using specimen dimensions of 100 mm × 100 mm × 3 mm.

TGA coupled with infrared spectroscopy (TGA–IR) was performed using an STA 2500 thermogravimetric analyzer (Netzsch, Selb, Bavaria, Germany) interfaced with a Thermo Fisher IS-50 FTIR spectrometer (Thermo Fisher, Waltham, MA, USA) at a heating rate of 10 °C/min in air to identify the volatiles formed during pyrolysis.

Raman spectra were recorded at room temperature using a LabRAM HR Evolution laser Raman spectrometer (Horiba Jobin Yvon Co., Kyoto, Kyoto Prefecture, Japan) with a 532 nm helium–neon laser.

X-ray photoelectron spectra (XPS spectra) were acquired on a Thermo Scientific K-Alpha instrument (Thermo Scientific Co., Oxford, Oxfordshire, UK) with an Al Kα source (1486.6 eV).

## 3. Results and Discussion

### 3.1. Structural Characterization

**Figure 2:**
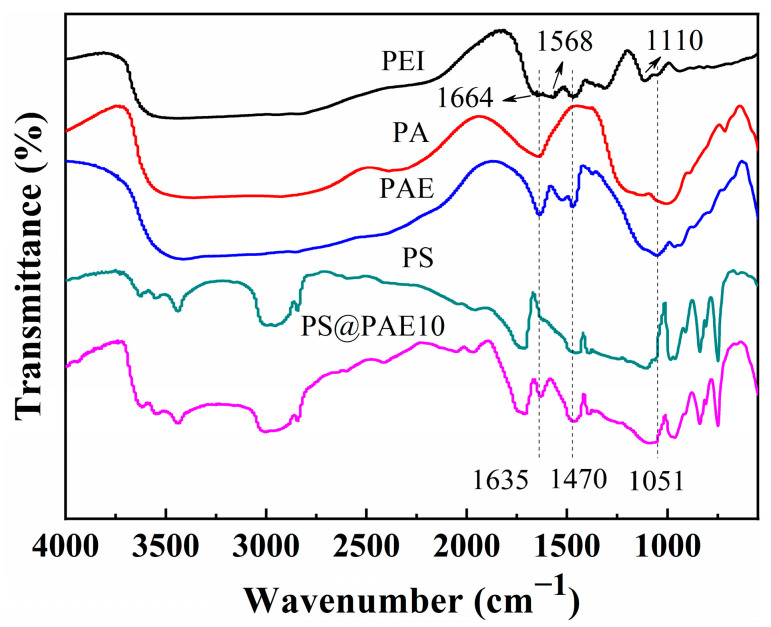
Fourier transform infrared (FTIR) spectra of PS, PA, PEI, PAE, and PS/PAE10.

PEI, PA, PAE, PS, and PS/PAE10 were examined using FTIR spectroscopy (Figure 2). The spectrum of PEI featured the peaks of –NH_2_ (1664 cm^−1^), –NH– (1568 cm^−1^), –CH_2_– (1470 cm^−1^), and C–N (1010 cm^−1^) groups, confirming the branched-chain nature of this material [42]. The spectrum of PA featured the peaks of P=O (1131 cm^−1^), and P–O (1003 cm^−1^) moieties [43,44]. The spectrum of PAE exhibited a characteristic amino group peak at 1560 cm^−1^. Additionally, a new signal at 1051 cm^−1^ was observed, which is attributed to ionic interactions between PA and PEI [28]. This suggests that PAE was formed via ion complexation between PA and PEI. The overlap between the PA and PEI peaks within the 1000–1300 cm^−1^ range resulted in enhanced signal intensities. The spectrum of PS/PAE10 displays characteristic signals of both PAE and PS, indicating that PAE retains its stable structure after hot-press molding. This stability is mainly attributed to the strong ionic and hydrogen bonds between PA and PEI, which ensure the chemical integrity of PAE during the process, indicating that PAE is compatible with the employed molding process.

### 3.2. Morphological Analysis

**Figure 3:**
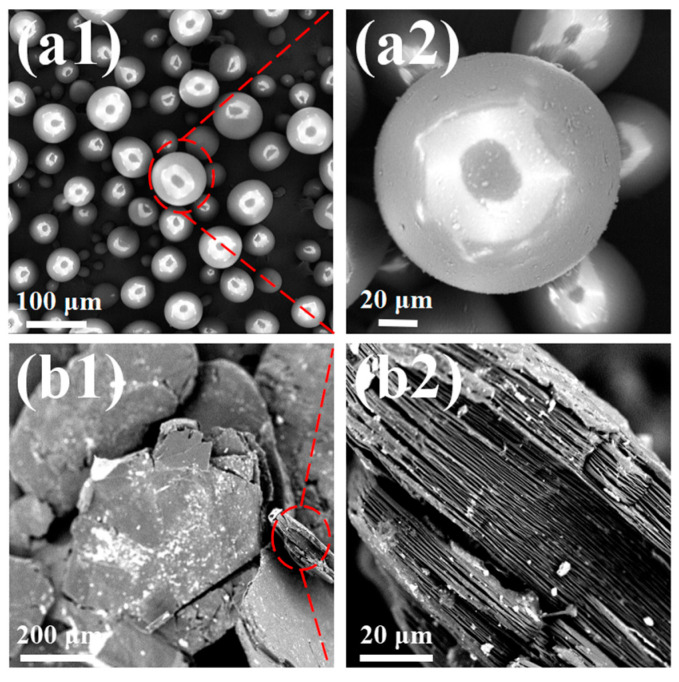
Scanning electron microscopy (SEM) images of (**a1**,**a2**) PS and (**b1**,**b2**) expandable graphite (EG).

SEM imaging (Figure 3) revealed that most PS microspheres had smooth surfaces and diameters of 40–80 μm, while EG featured an irregular flake-like structure with longitudinal and transverse dimensions exceeding 200 μm.

**Figure 4:**
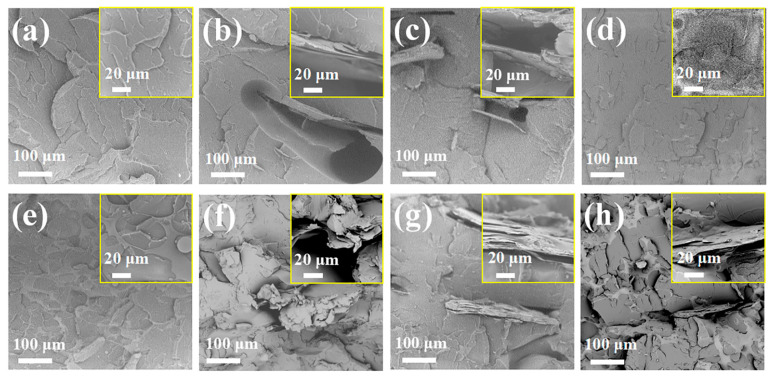
Cross-sectional SEM images of (**a**) PS, (**b**) PS/EG5, (**c**) PS/EG10, (**d**) PS/PAE5, (**e**) PS/PAE10, (**f**) PS/PAE3/EG7, (**g**) PS/PAE5/EG5, and (**h**) PS/PAE7/EG3.

The cross-sectional SEM images of PS and its composites (Figure 4) provided the following insights. PS (Figure 4a) showed a typical brittle fracture surface, whereas in PS/EG5 (Figure 4b) and PS/EG10 (Figure 4c), the EG layers were delaminated from the PS matrix, and large cracks and voids in the cross-sectional area were therefore observed. This delamination was attributed to the weak interfacial interaction between the EG layers and PS and was more pronounced for PS/EG10.

In contrast, in PS/PAE5 (Figure 4d) and PS/PAE10 (Figure 4e), PAE formed a thin flame-retardant adherent layer on the PS matrix, which was ascribed to the strong interfacial interaction between PAE and PS caused by hydrogen bonding between the primary and secondary amino groups of PEI and the polar (e.g., carbonyl and hydroxyl) groups on the PS surface. In the PS/PAE/EG composites (Figure 4f–h), the EG layers were closely attached to the PS matrix, which indicated that PAE enhanced the interfacial adhesion between EG and PS.

**Figure 5:**
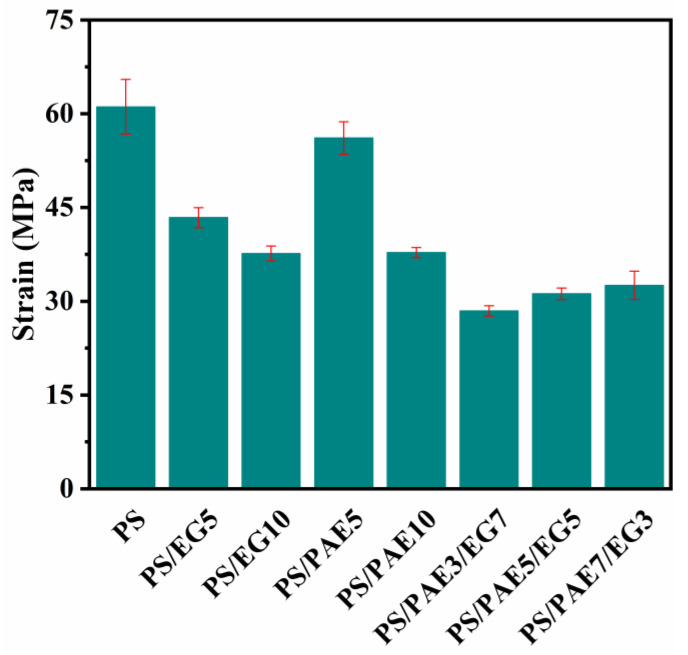
Tensile strengths of polystyrene and its composites.

Given that both PS and its composites exhibit brittle characteristics with elongation at break ranging from 2% to 4%, the analysis was confined exclusively to tensile strength. The tensile strengths of the various composites (Figure 5) indicate that the incorporation of EG leads to a deterioration in mechanical performance. This decline can be attributed to the inherently weak interlayer bonding and increased interlayer spacing of EG, which compromises the mechanical integrity of the composites. These structural weaknesses promote the formation of interfacial defects and voids within the polymer matrix, acting as stress concentrators and ultimately reducing the overall mechanical strength [45]. The tensile strength of PS/PAE5 is 56.1 MPa (91.8% of PS’s 61.1 MPa), while PS/PAE10 drops to 37.8 MPa (61.9% of PS). This decrease likely stems from defects and stress concentrators in the polymer matrix caused by PAE. When PAE and EG are used together for flame retardancy, the tensile strength of the composite remains slightly above 30 MPa, indicating a certain balance in mechanical properties. However, compared to PS/PAE5, the strength is still significantly reduced, likely due to the further weakening of interfacial bonding by EG, which decreases stress transfer efficiency. Despite this, the combination shows excellent flame-retardant performance. Future research could explore surface modification or compatibilizer addition to improve mechanical properties.

### 3.3. Thermal Stability and Char Analysis

**Figure 6:**
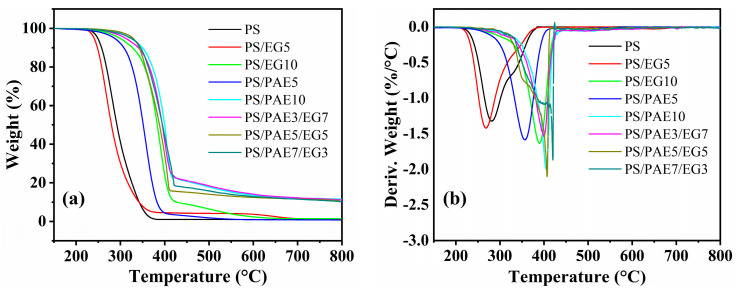
(**a**) Thermogravimetry and (**b**) differential thermogravimetry curves of PS and its composites.

Figure 6 displays the TGA and differential thermogravimetry curves of pure PS and its composites. Table 2 lists the parameters extracted from these curves, namely the initial decomposition temperature (T_onset_, temperature at 5% weight loss), temperature corresponding to the maximum heat loss rate (T_max_), and residue at 800 °C.

The T_onset_ and T_max_ of pure PS were 247 and 281 °C, respectively, with complete decomposition occurring above 400 °C. Owing to the low thermal stability of EG, the T_onset_ and T_max_ of PS/EG5 decreased to 240 and 268 °C, respectively. However, the T_onset_ and T_max_ of PS/EG10 were notably higher (298 and 390 °C, respectively). This thermal stability improvement was attributed to the formation of a protective thermally insulating carbon layer through the thermal expansion of a large amount of EG. It is worth noting that due to the rapid and significant thermal expansion of EG, it flew out of the thermogravimetric crucible, resulting in lower measured char residues of only 1.3% for PS/EG5 and 1.5% for PS/EG10.

Compared with EG, PAE exhibited a more pronounced flame-retarding effect on PS. The T_onset_ and T_max_ of PS/PAE5 were 286 and 357 °C, respectively, exceeding those of pure PS by 38 and 76 °C, respectively. Thus, the introduction of PAE enhanced the thermal stability of the polymer. The T_onset_ and T_max_ of PS/PAE10 were 321 and 405 °C, respectively, exceeding those of PS/EG10 by 23 and 15 °C, respectively. Additionally, the residue of PS/PAE10 after treatment at 800 °C was as high as 11.6%. This thermal stability improvement was ascribed to (i) the expansion effect associated with the decomposition of amino groups in PAE, which formed a flame-retardant base layer, and (ii) the formation of a thermally insulating polyphosphate layer through the dehydration condensation of phosphate groups in PAE, coupled with the excellent carbon-forming ability of PAE during thermal oxidation [28]. The PS/PAE/EG composites exhibited an even higher thermal stability due to the synergy between PAE and EG, showing T_onset_ > 310 °C, T_max_ > 400 °C, and residue > 10%. This enhancement was ascribed to the expansion effect of PAE decomposition and the viscosity of polyphosphoric acid effectively restraining the flying ash phenomenon of EG. The char layer effectively inhibited oxygen and heat transfer, thereby delaying the thermal degradation of the underlying materials.

**Figure 7:**
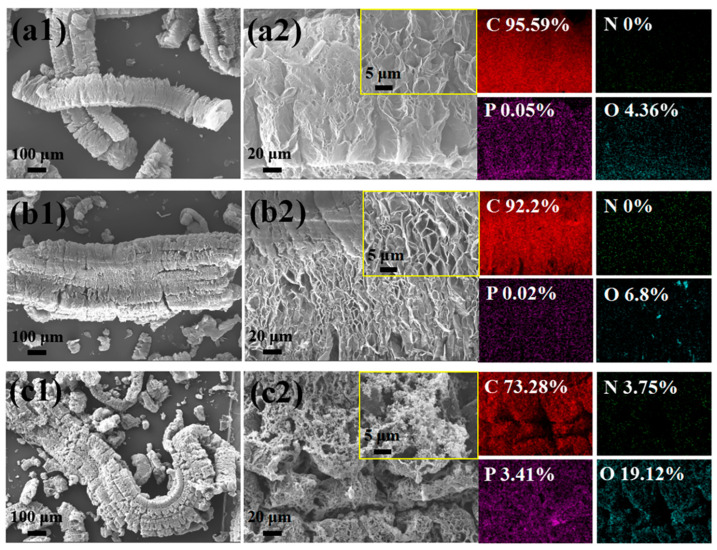
EDS elemental mappings of residues: (**a**) EG residues and (**a1**) magnified view with elemental mapping; (**b**) PS/EG10 residues and (**b1**) magnified view with elemental mapping; (**c**) PS/PAE5/EG5 residues and (**c1**) magnified view with elemental mapping.

**Figure 8:**
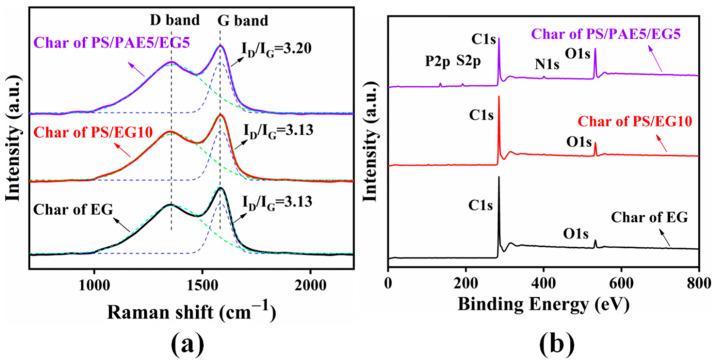
(**a**) Raman and (**b**) XPS spectra of the residual char produced from EG, PS/EG10, and PS/PAE5/EG5.

To further investigate the thermal properties of the composites at high temperatures, we placed PS, EG, PS/EG10, PS/PAE10, and PS/PAE5/EG5 (~1 g) in a high-temperature furnace and heated them at 800 °C for 5 min in air. Almost no residue formation was observed for PS and PS/PAE10. The microstructures and chemical compositions of these residues were investigated using SEM, energy-dispersive X-ray spectroscopy (EDS), Raman spectroscopy, and X-ray photoelectron spectroscopy (XPS). The SEM images and EDS data of the residues from EG, PS/EG10, and PS/PAE5/EG5 are presented in Figure 7, while the corresponding Raman and XPS spectra are depicted in Figure 8a and 8b, respectively.

The expanded graphite obtained through the thermal expansion of EG exhibited a worm-like porous microstructure (Figure 7(a1,a2)) and contained carbon (95.59%), minor amounts of oxygen (4.36%), and traces of phosphorus and nitrogen (0.05%). This indicates that the phosphorus in EG escaped at elevated temperatures. The residue of PS/EG10 was fluffy worm-like expanded graphite similar in morphology and composition to the EG residue (Figure 7(b1,b2)). This finding suggests that the thermal stability of PS/EG10 was due to the absorption of heat by EG and its expansion, which delayed heat ingress into the PS interior and thereby increased the thermal stability of PS.

The morphology and elemental composition of the residue from PS/PAE5/EG5 exhibited considerable differences. The worm-like expanded graphite surface was coated with nanoparticles primarily composed of phosphorus and nitrogen (Figure 7(c1,c2)). The residue contained 3.41% phosphorus, 3.75% nitrogen, and 19.12% oxygen. These results indicate that the polyphosphates generated by the thermal decomposition of PAE remained within the worm-like expanded carbon layer, augmenting its barrier effect.

The graphitization degree of residual carbon was quantified using Raman spectroscopy (Figure 8a), and the corresponding spectra featured the D (1372 cm^−1^) and G (1582 cm^−1^) bands of amorphous and graphitic carbon, respectively. The D/G band intensity ratio (*I*_D_/*I*_G_) is a quantitative measure of the graphitization degree [46]. The *I*_D_/*I*_G_ values of the chars obtained from PS and PS/EG10 were 3.13, indicating a relatively low degree of graphitization. This finding was attributed to the formation of worm-like expanded graphite because of the thermal expansion of EG, which created a porous network structure. The disruption of the original crystalline structure compromised the lattice integrity of graphite and thereby reduced the graphitization degree. Additionally, the expansion-blowing effect could have resulted in insufficiently high or sustained combustion temperatures, preventing the completion of graphitization [47].

The residues of EG, PS/EG10, and PS/PAE5/EG5 were probed by XPS to investigate the oxidation states of carbon therein. The residues of EG and PS/EG10 contained only carbon and oxygen, and therefore primarily originated from the worm-like graphite formed by the thermal expansion of EG (Figure 8b). In contrast, the PS/PAE5/EG5 residue also contained phosphorus and nitrogen. The P 2p peaks at 133.65, 134.95, 134.10, and 132.40 eV were ascribed to P=N, P–N, P–O, and P=O moieties, respectively. The N 1s signals at 400.50 and 399.40 eV were attributed to P=N and P–N bonds, respectively [21]. These results suggested that PAE participated in carbonization, forming a P–O–C crosslinked carbon layer that enhanced the flame retardancy in the condensed phase [48]. This conclusion was supported by the results of SEM analysis.

**Figure 9:**
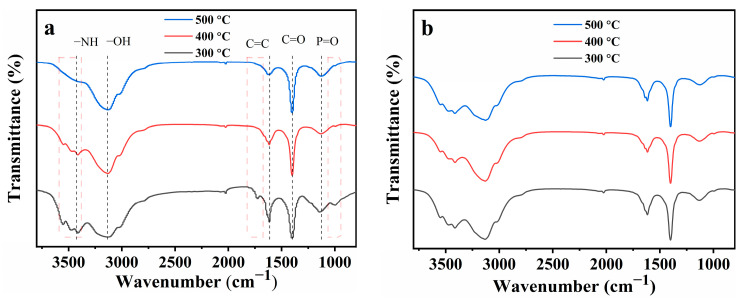
FTIR spectra of the (**a**) outer and (**b**) inner layers of PS/PAE5/EG5 residues obtained after combustion at different temperatures.

To further investigate the thermal degradation mechanism of the composites, we placed PS/PAE5/EG5 (~1 g) in a muffle furnace and heated it at 300, 400, or 500 °C for 5 min in air. The outer (Figure 9a) and inner (Figure 9b) layers of the resulting residues were analyzed by FTIR spectroscopy. As shown by the red dashed-line rectangles in Figure 9a, the signals near 3550, 3480, 3510, and 1730 cm^−1^ observed after heating at 300 and 400 °C indicated that the outer layer of the residue still contained PS (FTIR spectra of PS in Figure 2). The signal near 1000 cm^−1^ disappeared after heating at 400 °C, which suggested that the amino groups decomposed into volatile products between 300 and 400 °C. After heating at 500 °C, the PS peaks were almost undetectable. The broad peak near 3130 cm^−1^ was ascribed to the hydroxyl groups of phosphoric/polyphosphoric acid or ammonium salts [37]. The peak near 1620 cm^−1^ was attributed to the C=C moieties of benzene derivatives originating from the thermal degradation of PS [7]. The peaks at 1140 cm^−1^ were ascribed to the P=O groups of polyphosphoric acid produced by PA pyrolysis, respectively, indicating that phosphorus primarily exerted its flame-retardant effect in the condensed phase [29].

The absence of new peaks indicated the absence of notable reactions between PAE and EG during thermal degradation. The FTIR spectra of the inner layers of the residues exhibited no marked changes after heating at 300, 400, and 500 °C, indicating that (i) all composite components were stable and (ii) the expanded layer generated from the flame-retardant components upon heating effectively protected the PS matrix from thermal degradation, consistent with the TGA results.

**Figure 10:**
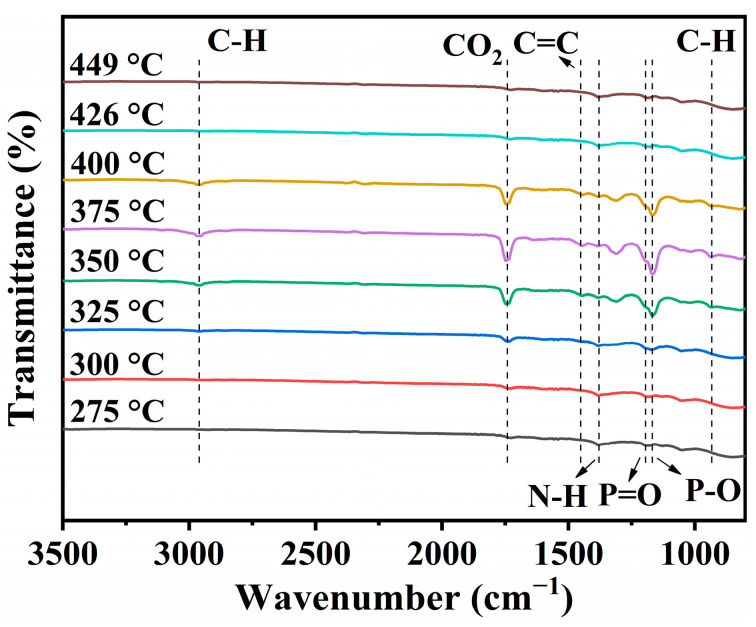
Thermogravimetric analysis–infrared spectroscopy profiles of the pyrolysis products of PS/PAE5/EG5 obtained at different temperatures.

The TGA–IR tests of PS/PAE5/EG5 (Figure 10) provided the following insights. The spectra recorded at ≥350 °C featured peaks at 2962 cm^−1^ (C–H stretching), 1454 cm^−1^ (C=C bending), 1168 (C–H bending), and 942 cm^−1^ (C=C bending) characteristic of the benzene ring of styrene, which is produced during the thermal decomposition of PS [2]. These signals reached their maximum intensities at 375 °C, which indicated that PS decomposition was most intense around this temperature. These peaks weakened at 400 °C and almost disappeared at 426 °C, which indicated that the pyrolysis range of PS was 375–426 °C, consistent with the TGA results.

The peaks at 1380 cm^−1^ (amine C–N stretching), 1195 cm^−1^ (P=O stretching), and 1149 cm^−1^ (P–O) observed at 275 °C essentially disappeared at 449 °C.

Based on the XPS spectra of EG-derived residual carbon (Figure 8b), the thermal expansion characteristics of the intercalation agent in EG, and the phosphorus content (Figure 7), it is inferred that the phosphorus-containing groups detected by TGA–IR originate from polyphosphates formed from PA due to the expansion effect of PEI and EG [49].

In conjunction with the TGA data and LOIs of PS/PAE5/EG5, these findings suggest that the polyphosphoric acid generated from PAE enhanced the heat barrier capability of char. This behavior confirms the formation of an effective thermal insulation due to the synergy between PAE and EG, which delayed the thermal decomposition of PS and notably improved its flame retardancy.

### 3.4. Flame-Retardant Properties and Burning Behaviors

**Figure 11:**
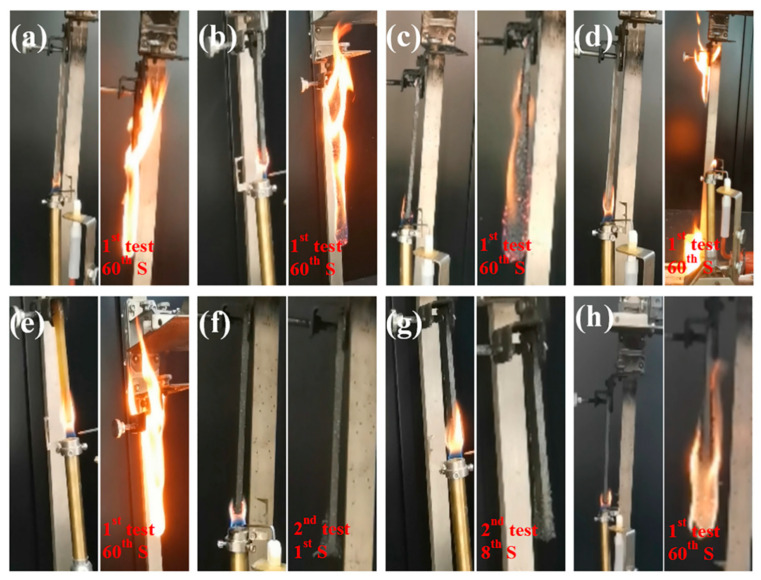
UL-94 burning photographs of (**a**) PS, (**b**) PS/EG5, (**c**) PS/EG10, (**d**) PS/PAE5, (**e**) PS/PAE10, (**f**) PS/PAE3/EG7, (**g**) PS/PAE5/EG5, and (**h**) PS/PAE7/EG3.

The results of the UL-94v test are shown in Figure 11 and Table 2. Table 2 also lists the LOIs. Pure PS featured a high LOI (17.2%), indicative of a high flammability, and exhibited dripping and cotton ignition during the UL-94v test (no rating; Figure 11a). The addition of PAE alone had a limited flame-retardant effect, with LOIs of 20.0% and 21.1% obtained for PS/PAE5 and PS/PAE10, respectively. EG showed better flame-retardant effects, with LOIs of 22.3% and 25.8% observed for PS/EG5 and PS/EG10, respectively. However, all these samples exhibited dripping and cotton ignition during the UL-94v test (no rating; Figure 11b–e). This poor performance was attributed to the easy destruction of the expanded graphite under the combined effects of thermal expansion and flame pressure, which led to the ineffective formation of a layer protecting against heat erosion and suppressing flammable gas volatilization.

The synergy between PAE and EG notably enhanced the flame-retardant performance of PS. PS/PAE3/EG7 achieved an LOI of 26.3% and passed the UL-94 V-0 rating (Figure 11f). PS/PAE5/EG5 showed the best performance, exhibiting an LOI of 27.7% and a V-0 rating (Figure 11g). PS/PAE7/EG3 had an LOI of 26.1%, but its burning time exceeded 60 s, and dripping and cotton ignition occurred during the UL-94v test (Figure 11h), resulting in an unrated performance. Thus, the optimal synergy between PAE and EG was achieved at a 1:1 mass ratio. This behavior was ascribed to the inhibitory effect of PAE on the flying ash phenomenon of EG, which resulted in a more flame-retardant carbon barrier layer in the condensed phase. The sticky polyphosphoric acid generated via PAE decomposition adhered to EG, suppressing the flying ash phenomenon and isolating oxygen and heat on the burning surface. Additionally, the nonflammable gas produced via PAE decomposition and that generated during the thermal expansion of EG reduced the oxygen concentration and temperature in the combustion area, effectively preventing the flame from spreading [48].

**Figure 12:**
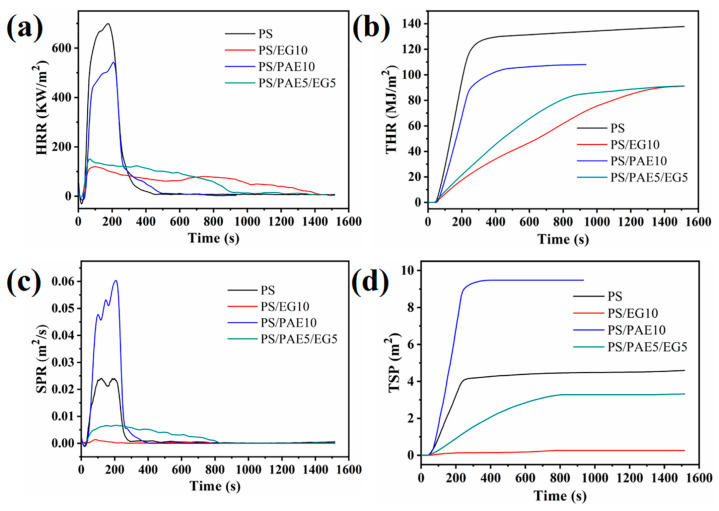
(**a**) Heat release rate, (**b**) total heat release, (**c**) smoke production rate, and (**d**) total smoke production curves of PS, PS/PAE10, PS/EG10, and PS/PAE5/EG5.

The flame-retardant performances of PS, PS/PAE10, PS/EG10, and PS/PAE5/EG5 were probed by the CCT. Figure 12 shows the HRR, THR, smoke production rate (SPR), and total smoke production (TSP) curves of PS and selected composites, with detailed data (including time to ignition (TTI), PHRR, THR, peak smoke production rate (PSPR), and residue mass loss rate) presented in Table 3.

The PHRR, THR, and TTI are critical parameters for evaluating the fire safety performance of materials, with lower values indicating better performance [50]. Pure PS had TTI = 36 s, PHRR = 716.4 kW/m^2^, THR = 138.2 MJ/m^2^, and mass loss rate = 0.16 g/s. After combustion, PS showed almost no residue, which indicated a rapid heat release and poor fire safety performance. The introduction of EG markedly improved the flame retardancy. PS/EG10 exhibited PHRR and THR values (121.7 kW/m^2^ and 91.0 MJ/m^2^, respectively) lower than those of pure PS by 83.0% and 34.2%, respectively. The SPR and TSP values of PS/EG10 were lower than those of PS by 93.7% and 94.4%, respectively, demonstrating an excellent smoke suppression performance. Smoke produced during a fire can be lethal, leading to suffocation and/or death due to the inhalation of toxic gases [51]. Therefore, EG plays a crucial role in improving the chances of escape and survival in a fire. The TTI of PS/EG10 (28 s) was lower than that of PS, and PS/EG10 showed a high residue content of 13.9%. This behavior indicated that EG did not initially protect PS during combustion, with its flame-retardant mechanism mainly involving rapid expansion at high temperatures to form a porous carbon layer. This layer effectively isolated the heat source, delaying and terminating polymer decomposition and achieving flame retardancy.

Compared with PS, PS/PAE10 exhibited a delayed TTI of 11 s, with PHRR = 539.9 kW/m^2^ and THR = 108.0 MJ/m^2^, representing reductions of 24.6% and 21.9%, respectively. The polyphosphoric acid generated during PAE combustion coated and protected the PS surface, leading to incomplete combustion and the release of NH_3_, N_2_, and other gases to favor the escape of incomplete combustion products [29,52]. Therefore, the PSPR and TSP of PS/PAE10 exceeded those of PS. PS/PAE10 had a carbon residue content of 3.3%, which indicated that PAE played a flame-retardant role in both the gas and condensed phases. Furthermore, substituting 5 phr of EG for the same amount of PAE in PS/PAE5/EG5 reduced the PHRR and THR (by 148.8 kW/m^2^ and 91.2 MJ/m^2^, respectively), which approached the values of PS/EG10. Owing to the inhibitory ability of EG, the PSPR and TSP of PS/PAE5/EG5 were 0.009063 m^2^/s and 3.3 m^2^/s, respectively, indicating a smoke suppression performance superior to those of PS and PS/PAE10. Additionally, the TTI of PS/PAE5/EG5 was delayed by 4 s, and the residue content (14.6%) exceeded that of PS/EG10. This indicated that EG and PAE exerted a synergistic effect when used together as flame retardants.

**Figure 13:**
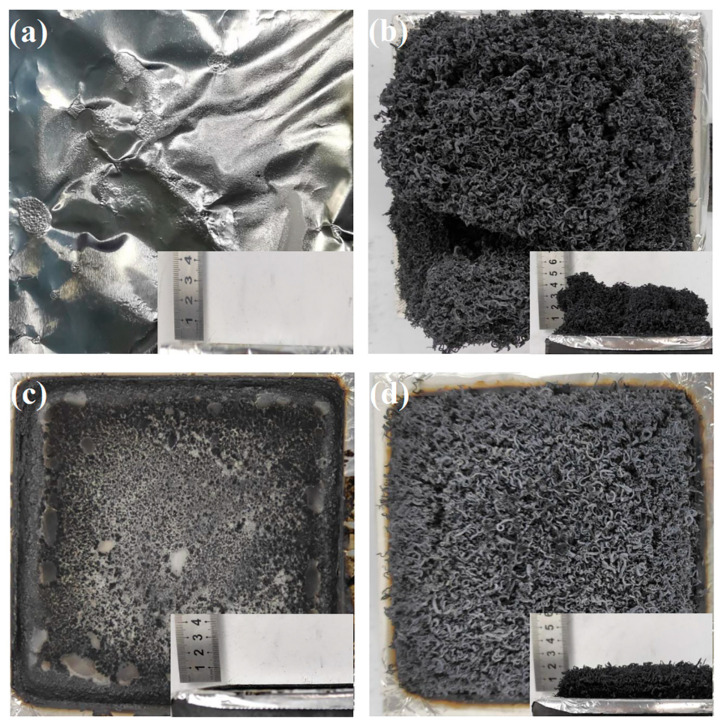
Images of char produced from (**a**) PS, (**b**) PS/EG10, (**c**) PS/PAE10, and (**d**) PS/PAE5/EG5 during CCTs.

Figure 13 presents the images of PS, PS/EG10, PS/PAE10, and PS/PAE5/EG5 residues produced after the CCT. PS burned completely with almost no residue, which indicated its high flammability (Figure 13a). PS/EG10 yielded a relatively loose carbon layer composed of worm-like expanded graphite produced during EG combustion (Figure 13b). The thickness of this char layer reached 4 cm.

PS/PAE10, which lacked carbon source components, produced only a small amount of residue after combustion (Figure 13c). Compared with PS/EG10, PS/PAE5/EG5 formed a denser and more stable residue (Figure 13d). Although the EG content of PS/PAE5/EG5 was lower than that of PS/EG10, the height of the PS/PAE5/EG5 residue (2 cm) was approximately half that of PS/EG10. The increased density of the PS/PAE5/EG5 residue was attributed to the interaction of polyphosphoric acid and the nitrogenous compounds generated during PAE combustion with the coke layer formed from EG. This interaction enhanced the stability and density of the coke layer. The high density and continuity of the char helped isolate heat and prevent the spread of oxygen and fuel, thereby markedly enhancing the condensed-phase flame-retardant effect [53,54].

### 3.5. Flame-Retardant Mechanism

**Figure 14:**
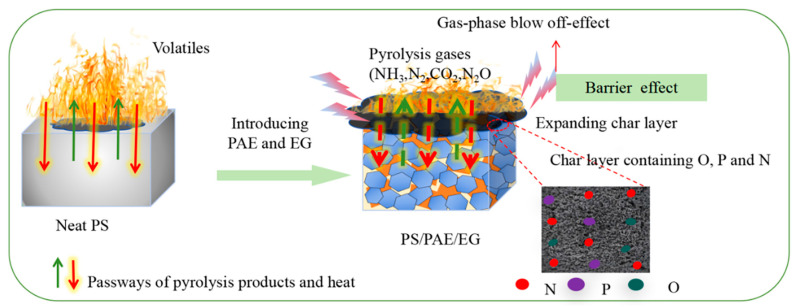
Flame-retardant mechanism of PS/PAE/EG composites.

Figure 14 presents the flame-retardant mechanism of the PS/PAE/EG composites based on the abovementioned results. Pure PS burns intensely because of its highly flammable nature. In contrast, the PS/PAE/EG composites exhibit a superior flame-retardant performance primarily attributed to the synergistic effects of PAE and EG in the condensed and gas phases.

In the condensed phase, EG rapidly expands upon heating to form a worm-like continuous and relatively dense expanded graphite layer adhering to the surface because of the presence of acid sources (e.g., polyphosphoric acid generated during PAE pyrolysis). This layer effectively suppresses the release of flammable gases/smoke and blocks the transfer of heat, oxygen, and other thermal degradation products to the surface, thereby protecting the PS matrix.

In the gas phase, the decomposition of the amine moieties of PAE generates inert gases (e.g., NH_3_, CO_2_, and N_2_), which dilute flammable volatiles and thereby reduce the combustion intensity. The rapid expansion of EG creates a near-vacuum state in the local space and exerts a blow-off effect, thus inhibiting flame propagation. Additionally, the endothermic decomposition of PAE and phase transition of EG during expansion absorb a considerable amount of heat, lowering the material temperature and further decreasing the HRR.

## 4. Conclusions

This study has successfully developed eco-friendly flame-retardant polystyrene (PS) composites by exploiting the synergistic effects of PA, PEI, and EG. The incorporation of PAE and EG into PS significantly enhanced the flame-retardant performance of the composites. The best-performing composite, with a PA:PEI:EG ratio of 1:1:1 (*w*/*w*/*w*) and a total flame-retardant loading of 10 parts per 100 parts of PS, exhibited an LOI of 27.7% and achieved a UL-94 V-0 rating. This composite demonstrated a substantial reduction in PHRR and THR, with decreases of 79.2% and 34.0%, respectively, compared to pure PS.

The strong interaction between EG and PAE provided an effective barrier against heat and oxygen, thereby improving the flame retardancy. The formation of a protective carbon layer and reduced HRR contributed to the enhanced flame-retardant performance of the composites. The presence of PAE and EG in the composites promoted the formation of a stable and dense char layer, which effectively isolated heat and prevented the spread of oxygen and fuel, thereby markedly enhancing the condensed-phase flame-retardant effect.

This study provides valuable insights into the development of high-performance, eco-friendly flame-retardant PS composites using PAE and EG. These flame retardants offer a sustainable alternative to traditional halogenated compounds. Future work should focus on optimizing formulations, enhancing mechanical properties, and exploring industrial applications. Further research could also investigate other renewable phosphorus sources and synergistic combinations to develop more effective flame-retardant systems. Overall, this study demonstrates the potential of PAE and EG to enhance the fire safety of PS materials, providing a foundation for future research in eco-friendly flame-retardant composites.

## Figures and Tables

**Table 1:** Formulations used for composite preparation (phr: parts per hundred parts of PS by weight).

Sample	PA (phr)	PEI (phr)	EG (phr)	PS (phr)
PS	0	0	0	100
PS/EG5	0	0	5	100
PS/EG10	0	0	10	100
PS/PAE5	2.5	2.5	0	100
PS/PAE10	5	5	0	100
PS/PAE3/EG7	1.5	1.5	7	100
PS/PAE5/EG5	2.5	2.5	5	100
PS/PAE7/EG3	3.5	3.5	3	100

**Table 2:** Parameters extracted from the thermograms of PS and its composites and results of the limiting oxygen index and UL-94v tests.

Samples	*T*_onset_ (°C)	*T*_max_ (°C)	Char Residue (%)	LOI (%)	UL94
PS	247	281	0.9	17.2	No rating
PS/EG5	240	268	1.3	22.3	No rating
PS/EG10	298	390	1.5	25.8	No rating
PS/PAE5	286	357	0.8	20.0	No rating
PS/PAE10	321	405	11.6	21.1	No rating
PS/PAE3/EG7	310	400	11.1	26.3	V-0
PS/PAE5/EG5	328	407	10.6	28.4	V-0
PS/PAE7/EG3	324	420	10.2	26.1	No rating

**Table 3:** CCT results for PS, PS/PAE10, PS/EG10, and PS/PAE5/EG5.

Parameters	PS	PS/PAE10	PS/EG10	PS/PAE5/EG5
TTI (s)	36	47	33	32
PHRR (W/m^2^)	716.4	539.9	361.6	148.8
THR (MJ/m^2^)	138.2	108.0	82.5	91.2
TSP (m^2^)	4.62	9.48	5.53	3.3
Av-EHC (MJ/kg)	28.6	30.7	22.8	30.9
Time of flameout (s)	440	470	1375	892
Residue thickness (cm)	0	0	4	2
Char residue rate (%)	0	3.3	13.9	14.6

## Data Availability

The original contributions presented in this study are included in the article. Further inquiries can be directed to the corresponding author.

## Supplementary Materials

The following supporting information can be downloaded at: https://www.mdpi.com/article/10.3390/ma18184308/s1, Figure S1: Calibration curve of absorbance versus concentration for Chlamydomonas solution; Table S1: Daily Absorbance and Concentration of Chlorella vulgaris in PAE and Control Groups [55,56].

## Abbreviations

The following abbreviations are used in this manuscript:

PS	Polystyrene
EG	Expandable graphite
PA	Phytic acid
PEI	Polyethyleneimine
PAE	Phytic acid–polyethyleneimine flame retardant
LOI	Limiting oxygen index
TGA	Thermogravimetric analysis
TGA–IR	Thermogravimetric analysis coupled with infrared spectroscopy
CCT	Cone calorimeter test
HRR	Heat release rate
PHRR	Peak heat release rate
SPR	Smoke production rate
PSPR	Peak smoke production rate
THR	Total heat release
TSP	Total smoke production
TTI	Time to ignition
