# Supplementary material for: Ecofriendly Flame-Retardant Polystyrene Composites: Exploiting the Synergistic Effects of Phytic Acid, Polyethyleneimine, and Expandable Graphite
Source: Materials (Basel). 2025 Sep 14;18(18):4308. doi: 10.3390/ma18184308 (PMC12471509; doi:10.3390/ma18184308)
Supplement: Supplementary file 1 [file materials-18-04308-s001.zip › materials-3855268-supplementary.pdf]

SUPPORTING INFORMATION

Ecofriendly flame-retardant polystyrene composites: Exploiting the synergistic effects of phytic acid, polyethyleneimine, and expandable graphite

Zhunzhun Li [1,2], Qimei Zhang [2], Jian Cui [1,*] and Yehai Yan [1,*]

[1] Key Lab of Rubber-plastics, Ministry of Education/Shandong Provincial Key Lab of Rubber-plastic, School of Polymer Science and Engineering, Qingdao University of Science and Technology, Qingdao 266042, China; lizz@czu.edu.cn
[2] Anhui Engineering Research Center of Highly Reactive Micro-Nano Powders, School of Materials and Environmental Engineering, Chizhou University, Chizhou 247000, China; zhangqm@czu.edu.cn
* Correspondence: jiancui@qust.edu.cn (J. C.), yhyan@qust.edu.cn (Y. Y.)

The PDF file includes:

Figure S1. Calibration curve of absorbance versus concentration for Chlamydomonas solution.

Table S1. Daily Absorbance and Concentration of Chlorella vulgaris in PAE and Control Groups.

This study aimed to assess the ecotoxicity of a flame retardant (PAE) synthesized from phytic acid and polyethyleneimine on the growth of Chlorella vulgaris.

1.1. Cultivation of Chlorella vulgaris [1,2]

0.1g of Chlorella vulgaris was inoculated into 250 ml of BG11 medium at a concentration of 1.5g/L.

The culture was maintained at 25±1 °C under a 14-hour light/10-hour dark cycle with a light intensity of 5000 lux for 5 days.

1.2. Preparation of PAE Treatment and Blank Control Groups

PAE Treatment Group: 20ml of the cultured Chlorella vulgaris suspension was added to 80ml of an aqueous dispersion containing 1g of PAE.

Blank Control Group: 20ml of the cultured Chlorella vulgaris suspension was added to 80ml of deionized water.

1.3. Measurement of Absorbance

The absorbance of Chlorella vulgaris was measured at a wavelength of 680nm using a spectrophotometer.

Measurements were taken daily over the 6-day period, and the absorbance data were recorded.

1.4. Establishment of Linear Relationship

Prior to the experiment, the linear relationship between the concentration of Chlorella vulgaris and absorbance at 685 nm was established by measuring the absorbance of Chlorella vulgaris suspensions at different concentrations.

This linear relationship $A = 0.0081*C + 0.0450$ was used to calculate the concentration of Chlorella vulgaris from the absorbance measurements. where A is the absorbance and C is the concentration of Chlorella vulgaris (mg/L).

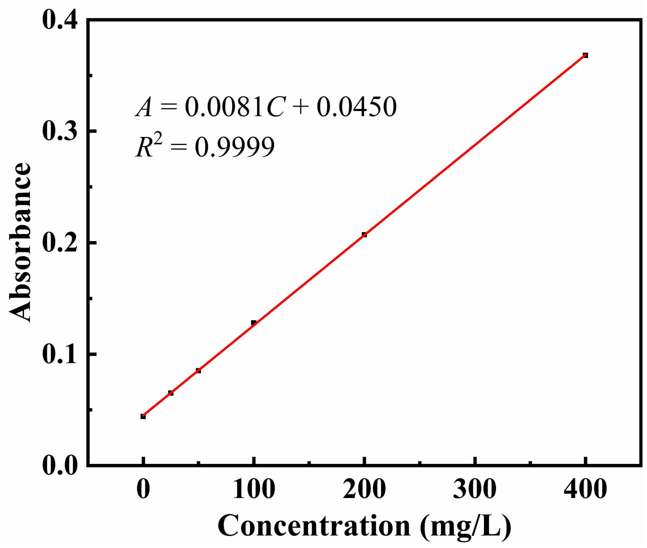

Figure S1. Calibration curve of absorbance versus concentration for Chlamydomonas solution

Table S1: Daily Absorbance and Concentration of Chlorella vulgaris in PAE and Control Groups.

Sample	Day 1	Day 2	Day 3	Day 4	Day 5	Day 6
Blank Control	0.093	0.091	0.075	0.074	0.066	0.058
PAE	0.105	0.143	0.136	0.137	0.134	0.135

2. Experimental Results

The results showed that PAE initially promoted the growth of Chlorella vulgaris, likely due to the additional nutrients provided by the phosphorus and nitrogen components in PAE. From day 3 onwards, the concentration of Chlorella vulgaris in the PAE treatment group stabilized, while that in the blank control group continued to decline. This suggests that PAE can maintain the growth of Chlorella vulgaris to some extent. This outcome may be related to the nutritional supplementation effect of the phosphorus and nitrogen in PAE on the growth of Chlorella vulgaris. Considering the overall experimental results, the synthesized PAE promotes the growth of Chlorella vulgaris in the short term and does not exhibit significant toxicity during the experimental period, indicating its good environmental compatibility. Therefore, PAE not only exhibits excellent flame-retardant properties in polystyrene composites but also shows good environmental compatibility in ecotoxicity tests, making it a promising ecofriendly flame retardant.

References

1. Lu, S.; Chen, B.; Yin, L.; Hao, X.; Ding, J.; Wang, X.; Wang, C.; Mei, R.; Wu, Y.; Zhang, H. The inhibiting effect on inhibition of chlorella growth by liverwort aqueous extract, *China Environ. Sci.* **2020**, *40*, 824-831. https://doi.org/10.19674/j.cnki.issn1000-6923.2020.0143.
2. Jia, Y.; Ma, Y.; Zheng, R.; Guo, J.; Zhang, L. Evaluation of biotoxicity changes in dye wastewater treated with Fe^0 /H_2O_2 using Chlorella vulgaris, *China Environ. Sci.* **2025**, *45*, 4322-4332. https://doi.org/10.19674/j.cnki.issn1000-6923.20250509.006.
